# Antiviral Activity of Baicalin Against PRRSV In Vitro and in Infected Piglets

**DOI:** 10.3390/vetsci13090915

**Published:** 2026-09-06

**Authors:** Zhiping Lu, Lei Xu, Dike Jiang, Ruijiao Jiang, Tianxiang Chen, Chunlin Fang, Hongli Jiang, Zili Li

**Affiliations:** 1 Key Laboratory of Preventive Veterinary Medicine of Hubei Province, College of Veterinary Medicine, Huazhong Agricultural University, Wuhan 430070, China; zplu@cdnkxy.edu.cn; 2 College of Animal Science and Veterinary Medicine, Chengdu Agricultural College, Chengdu 611130, China; xu_lei2013@163.com (L.X.); Jiangdk@cdnkxy.edu.cn (D.J.); jiangruijiaocac@cdnkxy.edu.cn (R.J.); chentx0219@163.com (T.C.); fangchunlin05@126.com (C.F.); jjiangmimi@126.com (H.J.)

**Keywords:** PRRSV, baicalin, viral attachment

## Abstract

Porcine reproductive and respiratory syndrome virus (PRRSV) causes substantial losses to the swine industry, creating a need for complementary antiviral interventions. This study evaluated the antiviral and protective effects of baicalin against PRRSV in vitro and in vivo. Cytotoxicity and antiviral activity were assessed in porcine alveolar macrophages (PAMs), and stage-specific assays were used to identify the affected phase of infection. Twenty piglets were assigned to Mock, baicalin, PRRSV, and PRRSV plus baicalin groups to evaluate clinical outcomes, viremia, respiratory shedding, tissue lesions, and inflammatory responses. Baicalin did not markedly affect PAM viability at concentrations up to 80 μg/mL. Within the tested non-cytotoxic concentration range, baicalin was associated with concentration-dependent reductions in infectious virus titers and PRRSV nsp9 mRNA levels. Among the stage-specific conditions examined, the greatest reduction in progeny virus production was observed when baicalin was present during the viral attachment phase, whereas no significant effects were detected in the direct virucidal, entry, intracellular replication, or progeny-release assays under the tested conditions. Compared with the PRRSV group, the PRRSV plus baicalin group showed lower viral loads in serum, oropharyngeal swabs, and nasal swabs at multiple sampling time points, together with less pronounced fever and clinical signs and partial preservation of body-weight gain. By the end of the experiment, four of five piglets survived in the PRRSV plus baicalin group, compared with one of five piglets in the PRRSV group. Serum IL-1β, IL-6, IL-8, and TNF-α concentrations were also lower in the PRRSV plus baicalin group at several sampling time points. Collectively, baicalin showed antiviral activity against PRRSV in PAMs, with the greatest activity observed when it was present during the viral attachment phase. In piglets, prophylactic dietary baicalin supplementation was associated with lower viral burden and less severe clinical, inflammatory, and pathological changes.

## Simple Summary

Porcine reproductive and respiratory syndrome is a major infectious disease that causes breathing problems, poor growth, reproductive losses, and death in pigs. Current vaccines do not always provide complete protection against the many virus strains circulating on farms, creating a need for additional control measures. Baicalin is a natural plant compound found in Chinese skullcap and has reported antiviral and anti-inflammatory properties. This study examined whether baicalin could inhibit the virus in porcine alveolar macrophages and protect infected piglets. Baicalin showed its greatest detectable antiviral activity when it was present during the stage in which the virus attaches to the cell surface. In piglets, dietary baicalin reduced the virus copies in the blood and decreased virus release in nasal and throat secretions. Compared with the PRRSV group, piglets receiving prophylactic dietary baicalin supplementation had less pronounced fever and clinical signs, better growth performance, less severe pulmonary and lymphoid lesions, and a numerically higher survival proportion. Baicalin also reduced several inflammatory substances associated with tissue injury. Overall, these results show that baicalin can interfere with an early stage of viral infection and reduce the severity of disease in piglets, supporting its further evaluation as a feed additive for porcine reproductive and respiratory syndrome control.

## 1. Introduction

Porcine reproductive and respiratory syndrome virus (PRRSV), first isolated as the Lelystad virus during outbreaks of “mystery swine disease” in Europe, is now recognized as one of the most consequential viral pathogens affecting global swine production [1]. In the United States, PRRSV-associated productivity losses were estimated at USD 664 million annually [2]. European field data likewise documented losses during an 18-week outbreak, with an average loss of EUR 126 per sow [3]. The disease is characterized by reproductive failure in sows, respiratory disease and growth retardation in young pigs, and prolonged disruption of innate and adaptive immunity, which increases susceptibility to secondary infections and amplifies production losses [4].

Vaccination, biosecurity, herd stabilization, and pathogen monitoring constitute the principal measures used to control PRRS. However, extensive genetic and antigenic diversity between and within PRRSV species limits heterologous vaccine protection, while the delayed and frequently weak neutralizing-antibody response further constrains vaccine efficacy against rapidly evolving field strains [5]. Modified-live virus vaccines generally provide stronger protection than inactivated vaccines, but their use is accompanied by concerns over residual replication, reversion or recombination, and transmission of vaccine-derived viruses; a recombinant derived from two vaccine strains caused significant production losses in Danish sow herds [6]. Host-directed approaches have demonstrated that CD163 is indispensable for productive PRRSV infection and that deletion of its scavenger receptor cysteine-rich domain 5 can confer resistance to both PRRSV genotypes [7,8]. Nevertheless, gene-edited animals cannot yet provide an immediate intervention for existing commercial herds. These limitations support the development of complementary compounds that either disrupt early virus–cell interactions or restrain the inflammatory injury that contributes to disease severity. Viral attachment and entry are particularly relevant targets because blocking these events can prevent infection before intracellular replication is established [9].

Baicalin is a flavone glucuronide and one of the principal bioactive constituents of the roots of *Scutellaria baicalensis* Georgi. Pharmacological studies have attributed antiviral, antioxidant, and immunoregulatory activities to this compound [10]. Baicalin inhibits influenza A virus-associated autophagy [11], interferes with dengue virus attachment and intracellular replication [12], reduces respiratory syncytial virus infection and virus-associated lung inflammation [13], and suppresses hepatitis B virus replication through tripartite motif-containing protein 25-dependent antiviral responses [14]. Its anti-inflammatory activity has been linked to suppression of nuclear factor kappa B and mitogen-activated protein kinase signaling and inhibition of the NOD-like receptor family pyrin domain-containing 3 inflammasome in cellular and animal models [15,16,17]. These pathways are relevant to PRRSV pathogenesis because infection of porcine alveolar macrophages induces mitochondrial dysfunction, cytosolic mitochondrial DNA stress, nuclear factor kappa B activation, and NOD-like receptor family pyrin domain-containing 3 inflammasome assembly, thereby promoting interleukin-1β production and inflammatory tissue injury [18]. Evidence specific to PRRSV, however, remains incomplete. Chang et al. demonstrated that baicalin inhibited several PRRSV strains in vitro and proposed direct virion interaction, modulation of CD151 and CD163 expression, and effects at multiple stages of infection [19]. Related work showed that the aglycone baicalein can bind PRRSV, target epidermal growth factor receptor signaling, and protect infected piglets [20]. A recent network pharmacology and metabolomics study further implicated metabolic and immune pathways in the in vitro activity of baicalin and Yinhuang soluble powder [21]. Despite this progress, the predominant stage of PRRSV infection affected by baicalin has not been independently resolved; its protective efficacy in infected piglets remains insufficiently established, and the relationship between its antiviral and anti-inflammatory effects in vivo is unclear.

The present study therefore aimed to systematically evaluate the antiviral and anti-inflammatory activities of baicalin against PRRSV in primary porcine alveolar macrophages and experimentally infected piglets and to define the stage of the viral life cycle most susceptible to treatment. We hypothesized that baicalin protects against PRRSV infection through a dual mechanism involving interference with viral attachment and attenuation of the subsequent pro-inflammatory response. By integrating stage-specific antiviral assessment with virological, clinical, pathological, and inflammatory outcomes, this study sought to establish an experimental basis for the development of baicalin as an adjunct intervention for PRRS control.

## 2. Materials and Methods

### 2.1. Cells and Virus

Porcine alveolar macrophages (PAMs) and the PRRSV SCSN2020 strain were isolated and maintained by the Animal Disease Diagnostic Center of Chengdu Agricultural College. SCSN2020 is a highly pathogenic PRRSV-2 strain.

### 2.2. Cell Viability Assay

Baicalin was dissolved in dimethyl sulfoxide (DMSO), sterilized through a 0.22-μm filter, and diluted in culture medium containing 0.1% DMSO to final concentrations of 10, 20, 40, 60, 80, 100, 200, 300, 400, and 500 μg/mL. The final DMSO concentration was maintained at 0.1% in all baicalin-treated wells. Mock wells contained PAMs cultured in medium containing 0.1% DMSO without baicalin, whereas untreated cell-control wells contained PAMs cultured in medium without baicalin or DMSO. Eight replicate wells were included for each condition. Blank wells containing culture medium without cells were also included. After incubation at 37 °C for 48 h, 10 μL of CCK-8 reagent was added to each well. Following a 2 h incubation in the dark, absorbance at 450 nm (OD450) was measured and cell viability was calculated using the equation shown below:

$$\mathrm{Cell}\;\mathrm{viability}\;\mathrm{inhibition}\;\mathrm{rate}\;\left(\%\right)=(1-\frac{{\mathrm{OD}}_{450}\mathrm{treatment}-{\mathrm{OD}}_{450}\mathrm{blank}}{{\mathrm{OD}}_{450}\mathrm{mock}-{\mathrm{OD}}_{450}\mathrm{blank}})\times 100\%$$

### 2.3. Quantitative Reverse-Transcription PCR

Total RNA was extracted from cells, tissues, and swab samples according to the RNAiso Plus protocol. RNA concentration and purity were measured using a ScanDrop spectrophotometer (Analytik Jena AG, Jena, Germany), and cDNA was synthesized using the PrimeScript RT Kit (Takara Biotechnology (Beijing) Co., Ltd., Beijing, China). qPCR was used to determine PRRSV copies and the expression levels of PRRSV nsp9. Relative gene expression was calculated using the 2^−ΔΔCt^ method with β-actin as the reference gene. Primer sequences are listed in Table 1.

### 2.4. In Vitro Anti-Prrsv Activity of Baicalin

(1) Prophylactic effect of baicalin

PAMs were seeded in 12-well plates and cultured to confluence. Baicalin was diluted to 80 μg/mL in RPMI 1640 containing 0.1% DMSO and 10% FBS. After three PBS washes, treatment wells received medium containing 80 μg/mL baicalin, and controls received RPMI 1640 containing 10% FBS and 0.1% DMSO without baicalin. Cells were incubated for 4 h at 37 °C with 5% CO_2_, washed three times, and inoculated with PRRSV at a multiplicity of infection (MOI) of 0.1. After 1 h, the inoculum was removed, and cells were washed three times before addition of 1 mL RPMI 1640 containing 2% FBS. After 24 h at 37 °C, samples were subjected to three freeze–thaw cycles and collected for virus titration.

PAMs were seeded into 96-well plates and cultured until a confluent monolayer was formed. PRRSV-containing samples were serially diluted 10-fold from 10^−1^ to 10^−8^ in RPMI 1640 medium. For each dilution, 25 μL of the diluted virus suspension was inoculated into each of eight replicate wells. Eight cell-control wells received 25 μL of virus-free RPMI 1640 medium. The plates were incubated at 37 °C with 5% CO_2_ for 1 h. The inoculum was then removed, and each well was supplemented with 100 μL of RPMI 1640 medium containing 2% fetal bovine serum. The plates were subsequently incubated at 37 °C with 5% CO_2_ for 3 days. Cytopathic effects were examined and recorded daily using an inverted microscope. The 50% tissue culture infectious dose (TCID_50_) was calculated using the Reed–Muench method and expressed as log_10_ TCID_50_/mL [22,23]. Each virus titration was performed in 3 independent experiments, with eight technical replicate wells per dilution.

(2) Direct virucidal effect of baicalin

For the baicalin-treated group, PRRSV was diluted to an MOI of 0.1 in serum-free RPMI 1640 containing 80 μg/mL baicalin. For the mock group, PRRSV was diluted to the same MOI in serum-free RPMI 1640 containing 0.1% DMSO without baicalin. Virus preparations were preincubated for 2 h at 37 °C with 5% CO_2_ and then added to PAM monolayers for 1 h. After three PBS washes, cells received fresh RPMI 1640 containing 2% FBS. Samples were collected after 24 h following three freeze–thaw cycles and subjected to virus titration.

(3) Effect of baicalin on PRRSV attachment

PAM monolayers were precooled at 4 °C for 1 h. PRRSV at an MOI of 0.1 was added in serum-free RPMI 1640 either with 80 μg/mL baicalin in 0.1% DMSO or with 0.1% DMSO alone as the mock group, and plates were incubated for 1 h at 4 °C. After removal of the inoculum and three PBS washes, 1 mL of fresh RPMI 1640 containing 2% FBS was added. After 24 h at 37 °C, samples were collected following three freeze–thaw cycles for virus titration.

(4) Effect of baicalin on PRRSV entry

PAM monolayers were precooled at 4 °C for 1 h and inoculated with PRRSV at an MOI of 0.1 for 1 h at 4 °C to permit attachment. Cells were washed three times with ice-cold PBS. Serum-free RPMI 1640 containing 80 μg/mL baicalin was then added to treatment wells, whereas serum-free RPMI 1640 containing 0.1% DMSO without baicalin was added to mock group wells. Plates were shifted to 37 °C for 1 h. After three PBS washes, 1 mL of fresh RPMI 1640 containing 2% FBS was added. Samples were collected after 24 h following three freeze–thaw cycles for virus titration.

(5) Effect of baicalin on intracellular PRRSV replication

PAM monolayers were infected with PRRSV at an MOI of 0.1 for 1 h at 37 °C. Following three PBS washes, treatment wells received RPMI 1640 containing 2% FBS and 80 μg/mL baicalin, whereas mock group wells received the same medium containing 0.1% DMSO without baicalin. Cells were cultured for 24 h, subjected to three freeze–thaw cycles, and collected for virus titration.

(6) Effect of baicalin on PRRSV release

PAM monolayers were infected with PRRSV at an MOI of 0.1 for 1 h at 37 °C. After three PBS washes, RPMI 1640 containing 2% FBS was added, and cells were cultured for 24 h. The medium was then removed, cells were washed, and medium containing 80 μg/mL baicalin and 0.1% DMSO or vehicle-control medium containing 0.1% DMSO without baicalin was added. After an additional 2 h at 37 °C, samples were subjected to three freeze–thaw cycles and collected for virus titration.

### 2.5. Animal Experimental Design

Twenty healthy, 21-day-old female Yorkshire piglets, with initial body weights ranging from 4.55 to 5.60 kg, were obtained from a commercial pig farm in Sichuan Province, China. Before enrollment, all piglets were confirmed to be negative for PRRSV antigen and PRRSV-specific antibodies. The animals were allowed to acclimatize to the experimental environment for 3 days before the experiment. Piglets were housed in separate pens maintained at 28 ± 1 °C and 60 ± 5% relative humidity, with ad libitum access to feed and water. The piglets were randomly allocated to four groups (*n* = 5 per group): PRRSV plus baicalin, PRRSV, baicalin, and Mock. Each group was housed separately to prevent cross-contamination. Investigators performing RT-qPCR, ELISA, clinical scoring, and histopathological assessment were blinded to group allocation until the corresponding measurements had been completed. Piglets in the PRRSV plus baicalin group received feed supplemented with baicalin at 0.1 g/kg, beginning 2 days before viral challenge and continuing throughout the experiment. On day 0, piglets in both the PRRSV plus baicalin and PRRSV groups were intranasally challenged with 3 mL of the PRRSV SCSN2020 strain at 10^6^ TCID_50_/mL. Piglets in the PRRSV group received the standard diet without baicalin. The baicalin group received baicalin-supplemented feed at 0.1 g/kg without viral challenge, whereas the Mock group received standard feed. Clinical signs, mortality, and body weight were monitored throughout the experiment [24]. Blood, oropharyngeal swabs, and nasal swabs were collected on days 3, 7, 14, 17, and 21. Tissue and serum samples were collected from moribund or dead piglets, including lung, liver, kidney, hilar lymph node and spleen. All surviving animals were euthanized on day 26 and the same samples were collected. Aliquots were stored at −80 °C for downstream analyses or fixed in 4% paraformaldehyde for histopathology. Viral shedding and viremia were evaluated by qRT-PCR.

### 2.6. Histopathology

Tissues fixed in 4% paraformaldehyde were dehydrated through a graded ethanol series, paraffin-infiltrated, embedded, and sectioned at 4 μm. Sections were deparaffinized in xylene, rehydrated through graded ethanol, stained with hematoxylin and eosin (H&E), dehydrated, cleared, and mounted with neutral resin. Histopathological changes were examined and photographed using an Eclipse 50i microscope (Nikon Corporation, Tokyo, Japan).

### 2.7. Cytokine Measurements

IL-1β, IL-6, IL-8, and TNF-α concentrations in serum were measured using ELISA kits according to the manufacturers’ instructions purchased from Beijing Solarbio Science & Technology Co., Ltd. (Beijing, China).

### 2.8. Statistical Analysis

Statistical analyses were performed using GraphPad Prism version 9.5.1. For the in vivo experiments, the individual piglet was considered the experimental unit. For the in vitro experiments, independent experiments, rather than individual technical replicate wells, were considered the experimental units. Technical replicates were averaged within each independent experiment before statistical analysis. Comparisons between two independent groups were performed using a two-sided unpaired Student’s *t*-test. Comparisons among multiple groups were performed using one-way analysis of variance (ANOVA). Dunnett’s multiple-comparisons test was used when multiple treatment groups were compared with a single control group, whereas Tukey’s multiple-comparisons test was used when all groups were compared with one another.

Because clinical, gross-lesion, and histopathological scores were ordinal, they were analyzed using the Kruskal–Wallis test followed by Dunn’s multiple-comparisons test. Survival was analyzed using the Kaplan–Meier method. Deaths and euthanasia after piglets reached the predefined humane endpoint were treated as events, whereas animals that remained alive at the end of the experiment were right-censored. Survival curves were compared using the two-sided log-rank (Mantel–Cox) test, and the proportions of animals surviving to the end of the experiment were additionally compared using Fisher’s exact test.

## 3. Results

### 3.1. Cytotoxicity of Baicalin in PAMs

**Figure 1:**
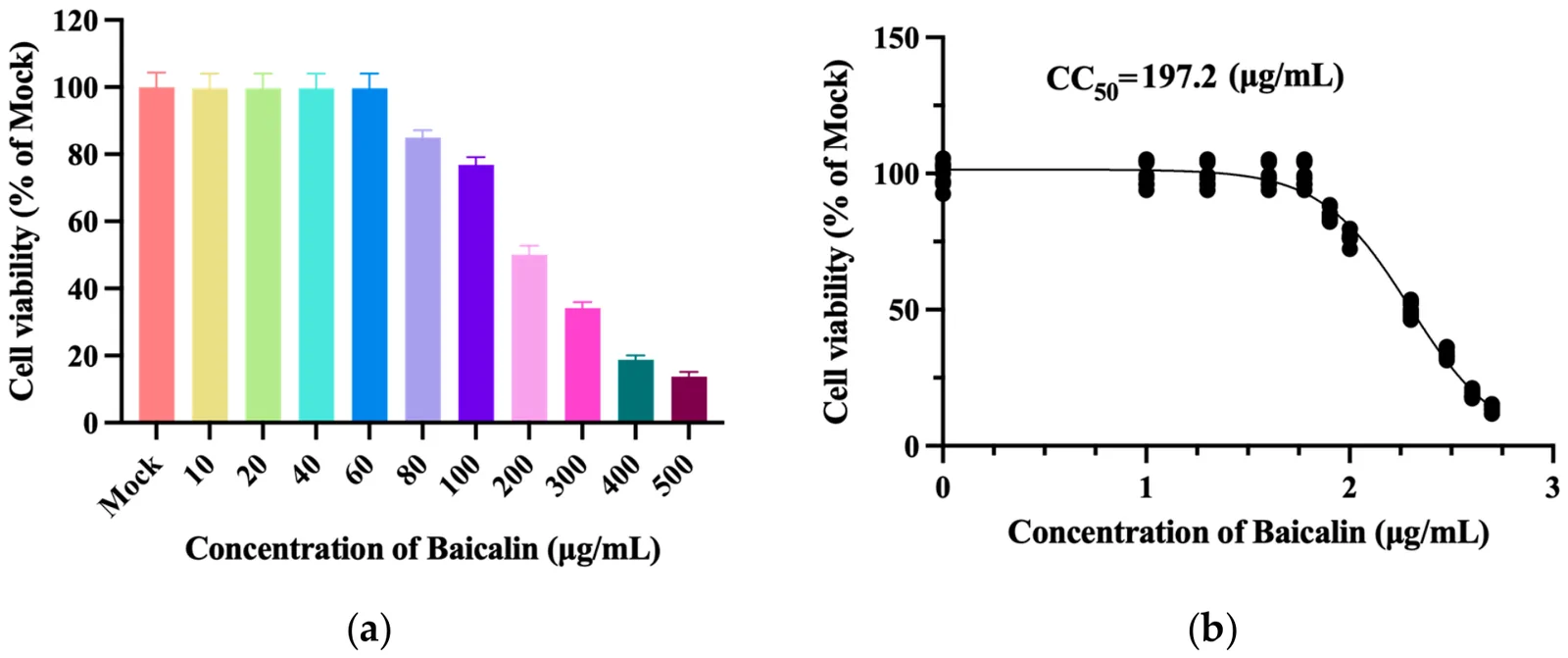
Effects of baicalin on PAM viability. (**a**) PAMs were treated with baicalin at 0 (Mock), 10, 20, 40, 60, 80, 100, 200, 300, 400, or 500 μg/mL for 48 h. Cell viability is expressed as a percentage of the Mock group. The different bar colors are used only to distinguish the tested baicalin concentrations and do not represent additional experimental variables. Data are presented as mean ± SD. (**b**) Cytotoxic concentration-response curve. Individual technical-well values are shown, and the curve was fitted using a four-parameter logistic regression model.

To define the non-cytotoxic concentration range of baicalin in PAMs, cell viability was measured using the CCK-8 assay after exposure to different concentrations of baicalin. Cell viability was normalized to that of the untreated control cells (containing neither baicalin nor DMSO), which was set at 100%. As shown in Figure 1, treatment with 10, 20, 40, 60, or 80 μg/mL baicalin for 48 h did not markedly affect PAM viability compared with the cell control, with viability remaining above 80%. At higher concentrations, cell viability progressively decreased; a clear reduction was observed at concentrations ≥200 μg/mL, with the greatest loss of viability in the 500 μg/mL group. A four-parameter logistic regression analysis of the concentration–response data yielded a CC_50_ of 197.2 μg/mL (Figure 1b).

### 3.2. Baicalin Inhibits PRRSV Infection In Vitro

**Figure 2:**
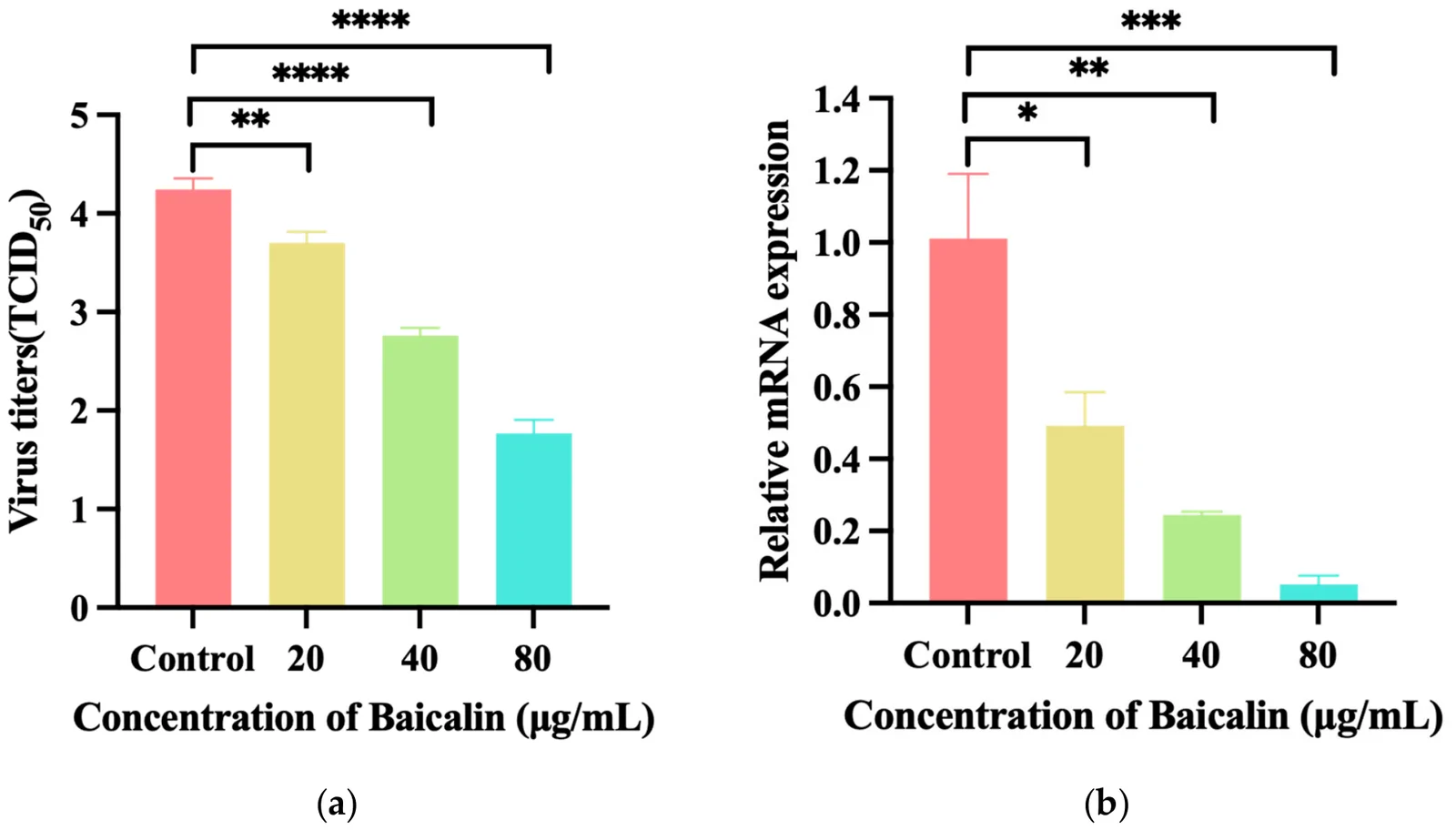
In vitro inhibitory effects of baicalin on PRRSV infection. PRRSV-infected PAMs were treated with the indicated concentrations of baicalin. (**a**) Infectious virus titers were determined by TCID_50_ assay. (**b**) Relative PRRSV nsp9 levels were determined by qRT-PCR. The different colors are used only to distinguish the PRRSV control group and the different baicalin concentration groups and do not represent additional experimental variables. Data are presented as mean ± SD. Compared with the PRRSV control group: * *p* < 0.05, ** *p* < 0.01, *** *p* < 0.001, **** *p* < 0.0001.

We next evaluated the antiviral effect of baicalin against PRRSV in PAMs. Virus titration showed that infectious titers remained high in the PRRSV control group but were significantly reduced after prophylactic dietary baicalin supplementation. Viral titers progressively decreased as the baicalin concentration increased from 20 to 80 μg/mL, with the lowest titer observed at 80 μg/mL (Figure 2a), indicating a concentration-dependent inhibitory effect on infectious virus production. Consistently, qRT-PCR showed that relative PRRSV nsp9 levels were markedly increased after infection but decreased following treatment with 20, 40, or 80 μg/mL baicalin, with more pronounced reductions at 40 and 80 μg/mL (Figure 2b). Together, these findings demonstrate that baicalin suppresses PRRSV infection in vitro in a concentration-dependent manner.

### 3.3. Baicalin Shows Its Greatest Antiviral Activity During the Attachment Phase of PRRSV Infection

**Figure 3:**
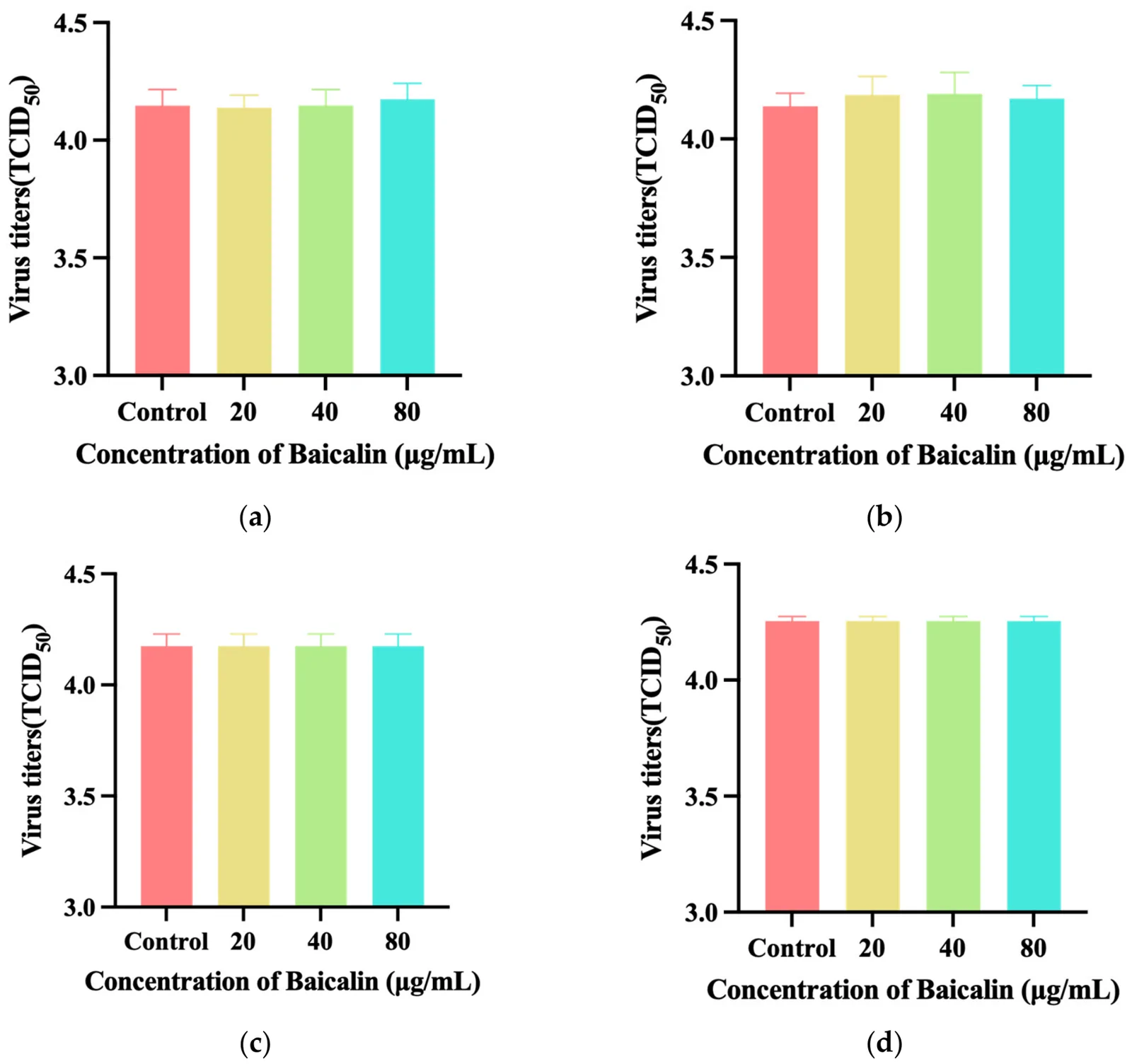

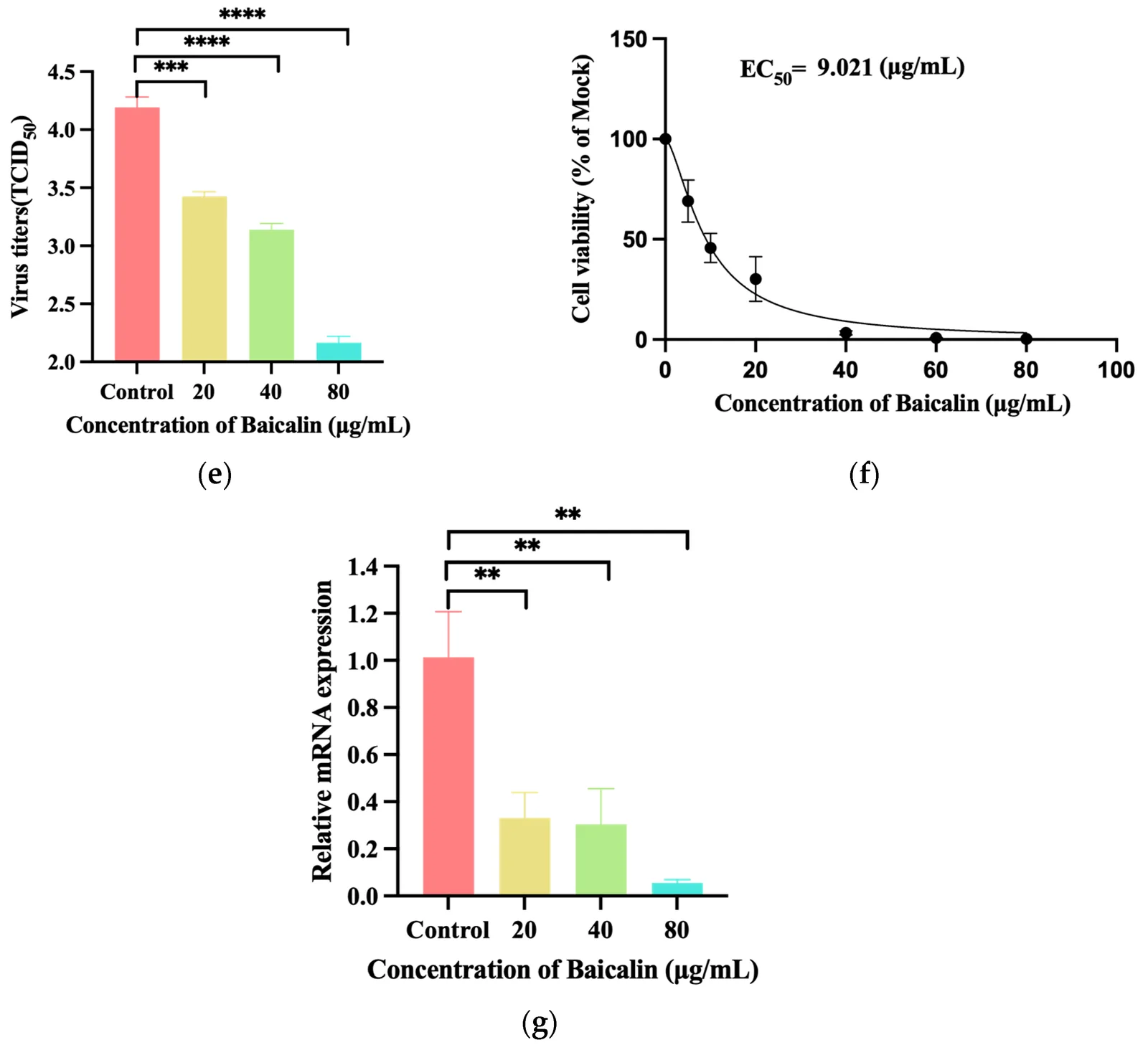
Effects of baicalin on different stages of the PRRSV life cycle. (**a**), viral entry (**b**), intracellular replication (**c**), virus release (**d**), or viral attachment (**e**,**g**). (**a**–**e**) Virus titers were determined by TCID_50_ assay. (**f**) Concentration–response curve generated using 5, 10, 20, 40, 60, and 80 μg/mL baicalin. Infectious virus yields were normalized to the vehicle-treated virus control, which was set at 100%, and fitted using a four-parameter logistic regression model. The calculated half-maximal effective concentration (EC_50_) was 9.021 μg/mL. (**g**) Relative PRRSV nsp9 levels after the attachment step were quantified by qRT-PCR. The different colors are used only to distinguish the virus control and the different baicalin concentration groups and do not represent additional experimental variables. Data are presented as mean ± SD. Compared with the virus control: ** *p* < 0.01, *** *p* < 0.001, **** *p* < 0.0001.

To identify the stage of PRRSV infection affected by baicalin, we separately evaluated direct virucidal activity, viral entry, intracellular replication, progeny release, and attachment. Preincubation of PRRSV with 20–80 μg/mL baicalin did not significantly alter subsequent virus titers relative to the virus control (Figure 3a), indicating no detectable direct virucidal activity under these conditions. Likewise, baicalin did not significantly affect virus titers when applied during viral entry (Figure 3b), intracellular replication (Figure 3c), or progeny release (Figure 3d).

In contrast, the greatest antiviral activity was observed when baicalin was present during the viral attachment phase. Subsequent infectious virus titers were significantly lower following exposure to 20, 40, or 80 μg/mL baicalin than in the virus control, and the magnitude of the reduction increased with baicalin concentration (Figure 3e), with the greatest reduction observed at 80 μg/mL. To further characterize the concentration–response relationship under this condition, nonlinear regression of infectious virus yields normalized to the vehicle-treated virus control yielded an EC_50_ of 9.021 μg/mL (Figure 3f). qRT-PCR analysis showed a similar concentration-dependent pattern, with significantly lower PRRSV nsp9 mRNA levels in all baicalin-exposed groups and the lowest level observed at 80 μg/mL (Figure 3g). These results indicate that, among the stage-specific conditions examined, the attachment phase was associated with the greatest detectable antiviral activity of baicalin.

### 3.4. Baicalin Alleviates Clinical Disease and Improves Survival in PRRSV-Infected Piglets

**Figure 4:**
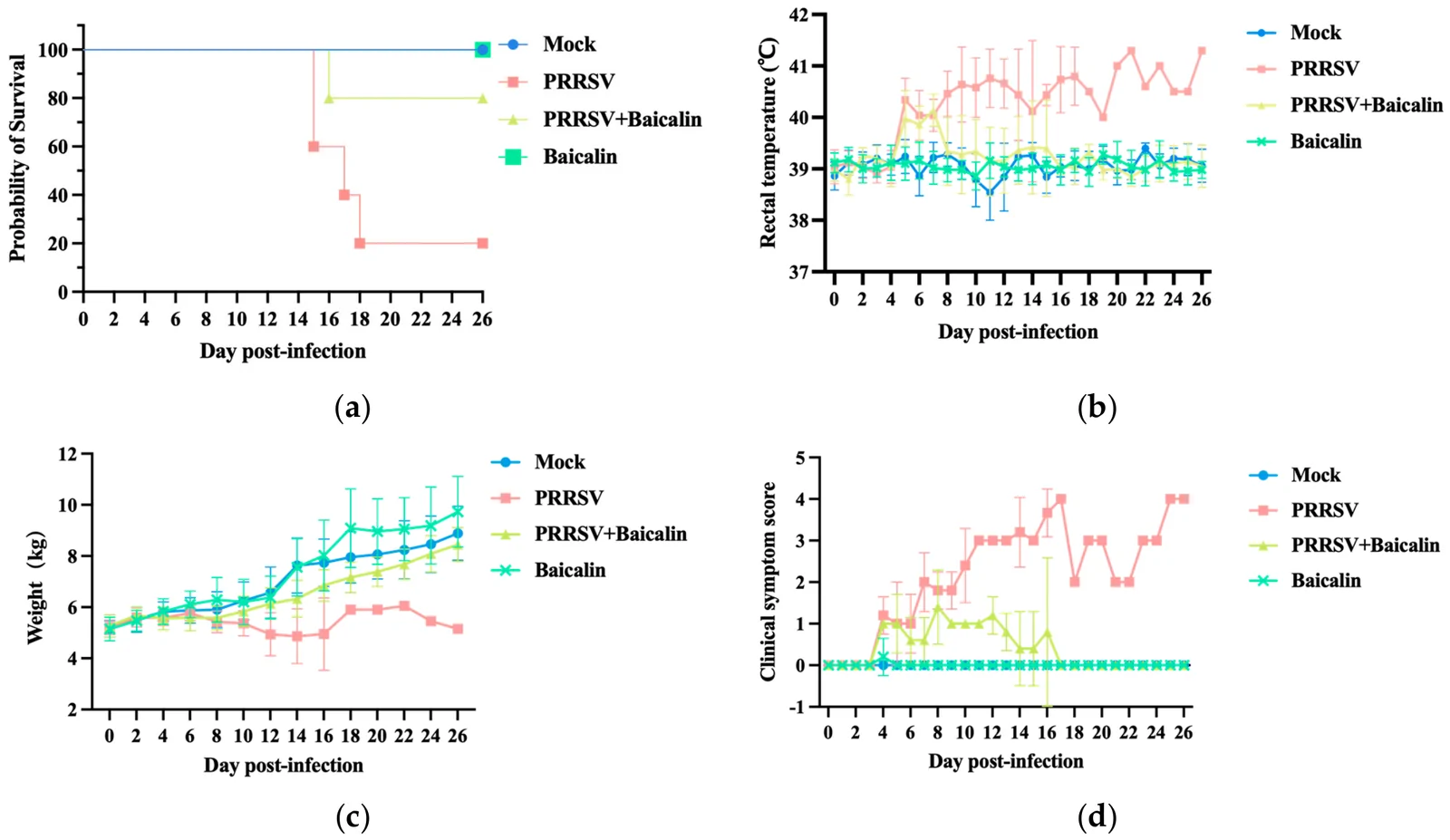
Effects of baicalin on clinical outcomes and survival in PRRSV-infected piglets. (**a**) Kaplan–Meier survival curves. (**b**) Rectal temperature after infection. (**c**) Changes in body weight. (**d**) Clinical symptom scores. Data are presented as mean ± SD where applicable.

To evaluate the protective effects of baicalin in vivo, survival, rectal temperature, body weight, and clinical scores were monitored throughout the experiment. All piglets in the Mock and baicalin-only groups survived. Mortality in the PRRSV group began on day 15 after challenge. Four of five piglets in the PRRSV group died during the observation period, whereas one of five piglets in the PRRSV plus baicalin group died. At the end of the experiment, the observed survival proportions were 20% and 80%, respectively (Figure 4a). However, the difference between the Kaplan–Meier survival curves did not reach statistical significance (log-rank test, *p* = 0.082). Likewise, the difference in the proportions surviving to day 26 was not significant according to Fisher’s exact test (*p* = 0.206).

Rectal temperatures in the Mock and baicalin groups remained within the normal range throughout the study (Figure 4b). In the PRRSV group, body temperature increased markedly from approximately day 5 after infection, remained above 40 °C for much of the observation period, and exceeded 41 °C at its peak. Piglets in the PRRSV plus baicalin group also developed a transient increase in body temperature, but the magnitude was lower and values generally remained between 39 and 40 °C. These findings suggest that baicalin attenuated PRRSV-associated persistent fever.

Initial body weights were comparable among groups (Figure 4c). Piglets in the Mock and baicalin groups gained weight steadily, whereas weight gain was markedly impaired after PRRSV infection and body weight tended to decline late in infection. In the PRRSV plus baicalin group, weight gain was relatively slow early after infection but gradually recovered during the middle and late stages. Thus, baicalin partially mitigated the adverse effect of PRRSV infection on growth performance.

Clinical scores remained low in the Mock and baicalin groups throughout the experiment (Figure 4d). Clinical signs appeared from day 4 after infection in the PRRSV group and reached their highest levels around days 15–16. Clinical signs were also observed in the PRRSV plus baicalin group, but overall scores were lower than those in the PRRSV group and gradually returned toward baseline during the late phase of infection. These data indicate that baicalin ameliorated clinical disease and promoted recovery after PRRSV infection. Collectively, dietary baicalin improved survival, reduced persistent fever and clinical severity, and alleviated impaired weight gain in PRRSV-infected piglets.

### 3.5. Baicalin Reduces Viremia and Respiratory Shedding in PRRSV-Infected Piglets

**Figure 5:**
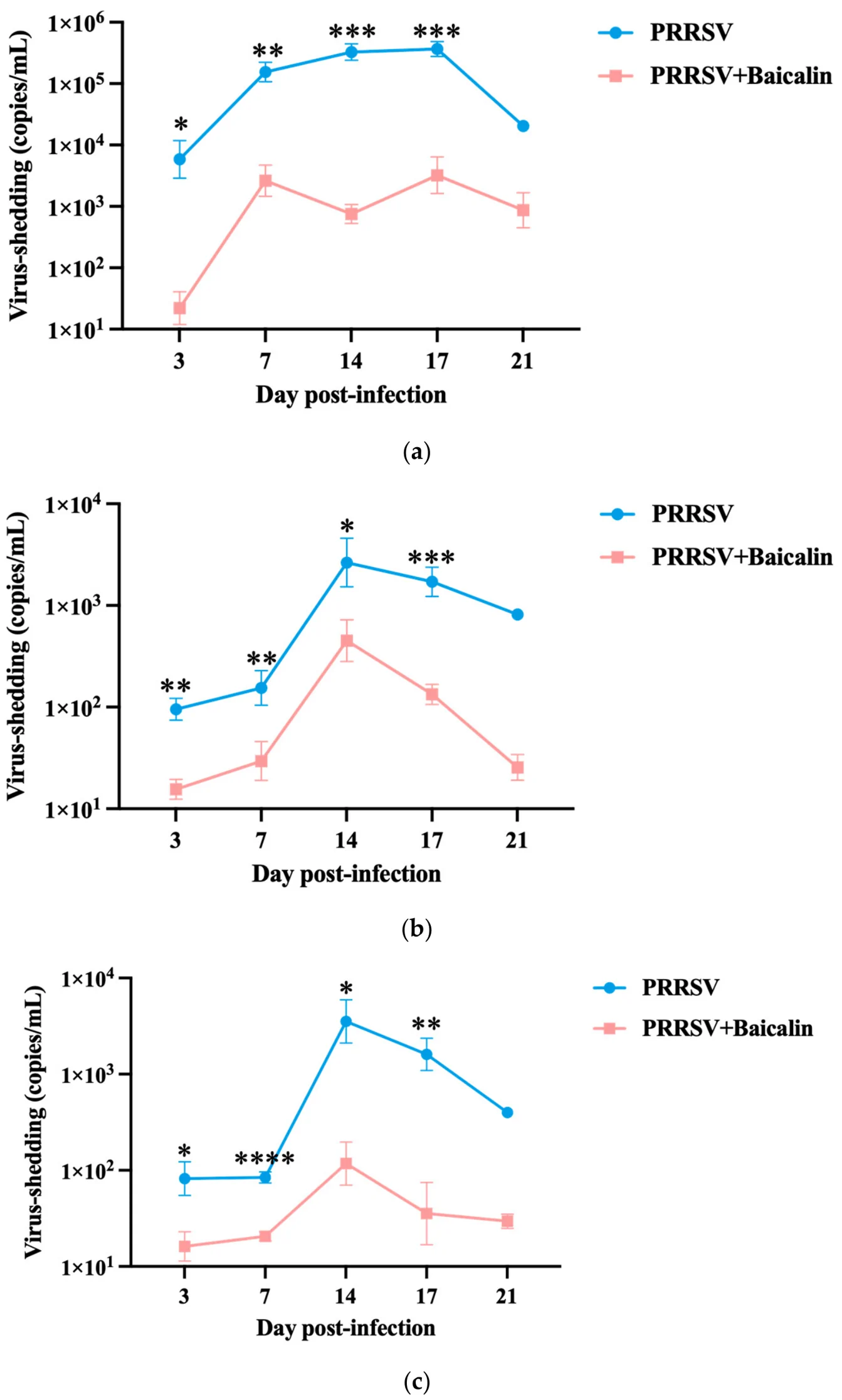
Effects of baicalin on viremia and viral shedding in PRRSV-infected piglets. PRRSV copies in (**a**) serum, (**b**) oropharyngeal swabs, and (**c**) nasal swabs. Data are presented as mean ± SD. Compared with the PRRSV group: * *p* < 0.05, ** *p* < 0.01, *** *p* < 0.001, **** *p* < 0.0001.

PRRSV copies in serum, oropharyngeal swabs, and nasal swabs were quantified by qRT-PCR to evaluate viral replication and shedding in vivo. High-level viremia was detectable in the PRRSV group by day 3 after infection and increased through days 14–17 before declining by day 21, although viral loads remained elevated (Figure 5a). Serum viral copies were consistently lower in the PRRSV plus baicalin group, with highly significant reductions on days 7, 14, and 17 and significant reductions on days 3 and 21.

PRRSV copies in oropharyngeal swabs increased after infection, peaked on day 14, and subsequently declined in the PRRSV group (Figure 5b). Viral loads in oropharyngeal swabs were lower in the PRRSV plus baicalin group. A similar pattern was observed in nasal swabs (Figure 5c): viral shedding was detectable from day 3, peaked on day 14, and then declined, whereas copies were consistently lower in infected piglets receiving prophylactic dietary baicalin supplementation. Together, these data show that baicalin reduced serum viral loads and decreased PRRSV shedding in both oropharyngeal and nasal secretions.

### 3.6. Baicalin Alleviates PRRSV-Induced Tissue Injury

**Figure 6:**
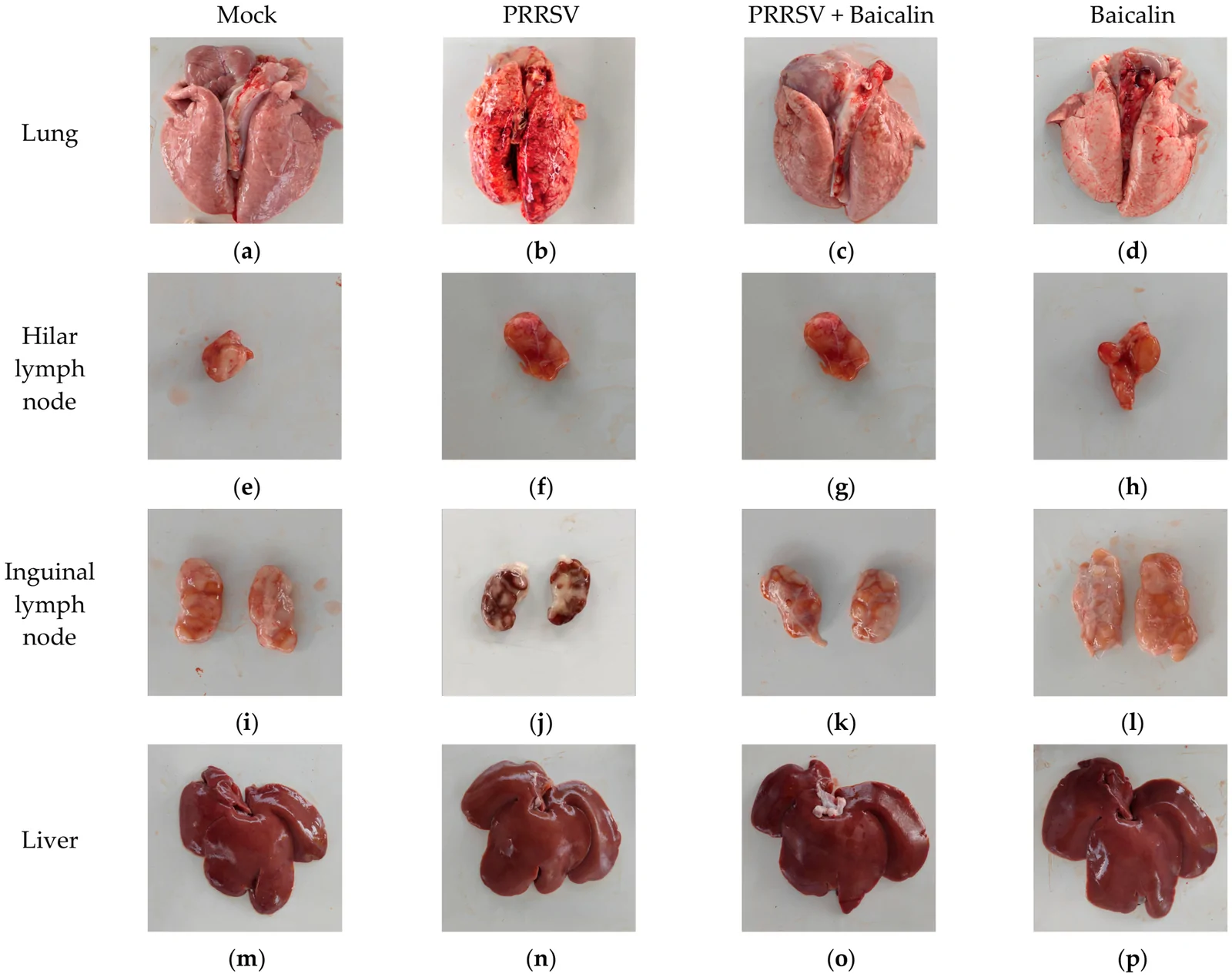

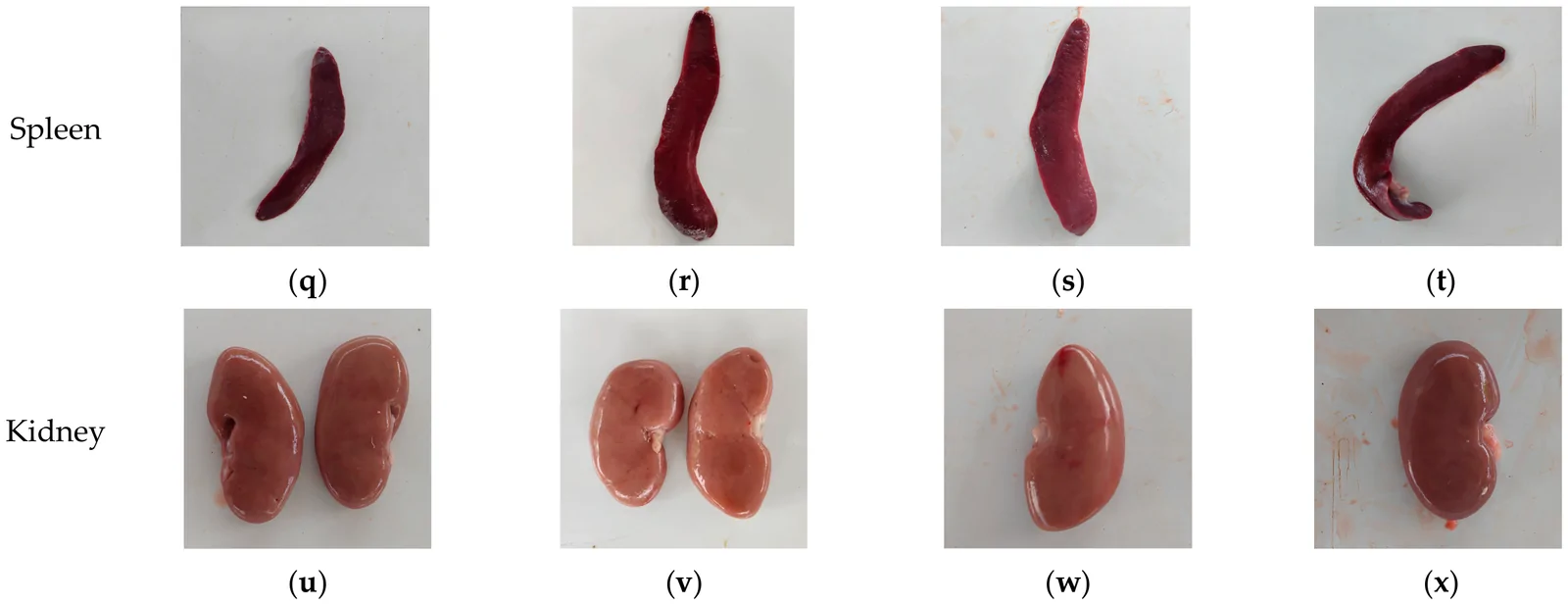
Representative gross pathological findings in major organs of PRRSV-infected piglets receiving prophylactic dietary baicalin supplementation. Representative gross appearances of the lung (**a**–**d**), hilar lymph node (**e**–**h**), inguinal lymph node (**i**–**l**), liver (**m**–**p**), spleen (**q**–**t**), and kidney (**u**–**x**) are shown. The four columns represent the Mock group (**a**,**e**,**I**,**m**,**q**,**u**), PRRSV group (**b**,**f**,**j**,**n**,**r**,**v**), PRRSV plus baicalin group (**c**,**g**,**k**,**o**,**s**,**w**), and baicalin-only group (**d**,**h**,**l**,**p**,**t**,**x**), respectively. Gross lesions were most pronounced in representative tissues from the PRRSV group, particularly in the lungs and lymph nodes, whereas these changes appeared less pronounced in the PRRSV plus baicalin group.

Gross pathological changes in the lungs, hilar lymph nodes, inguinal lymph nodes, liver, spleen, and kidneys were examined (Figure 6). In the Mock group, the lungs were uniformly pale pink with well-defined lobes and smooth surfaces, without obvious congestion, hemorrhage, or consolidation. Hilar and inguinal lymph nodes were relatively uniform in color and showed no obvious hemorrhagic or necrotic changes. The liver, spleen, and kidneys retained normal gross morphology. Tissues from the baicalin-only group were similar to those from the Mock group, with no obvious necrotic lesions.

In the PRRSV group, the lungs exhibited extensive dark-red discoloration, heterogeneous coloration, marked congestion and hemorrhage, and areas of consolidation. Hilar lymph nodes showed irregular reddish-brown discoloration, whereas inguinal lymph nodes contained prominent dark-red to brown-black lesions consistent with severe congestion, hemorrhage, or focal tissue injury. The spleen was also dark red and congested. No extensive hemorrhage or prominent necrotic foci were observed in the liver or kidneys.

Compared with the PRRSV group, PRRSV-infected piglets receiving baicalin showed less pulmonary congestion, hemorrhage, and consolidation. Overall lobar architecture was better preserved, although focal red lesions and mild congestion remained in some areas. Hilar and inguinal lymph nodes lacked the prominent dark hemorrhagic lesions observed in PRRSV group, and the liver, spleen, and kidneys showed relatively mild gross changes.

**Figure 7:**
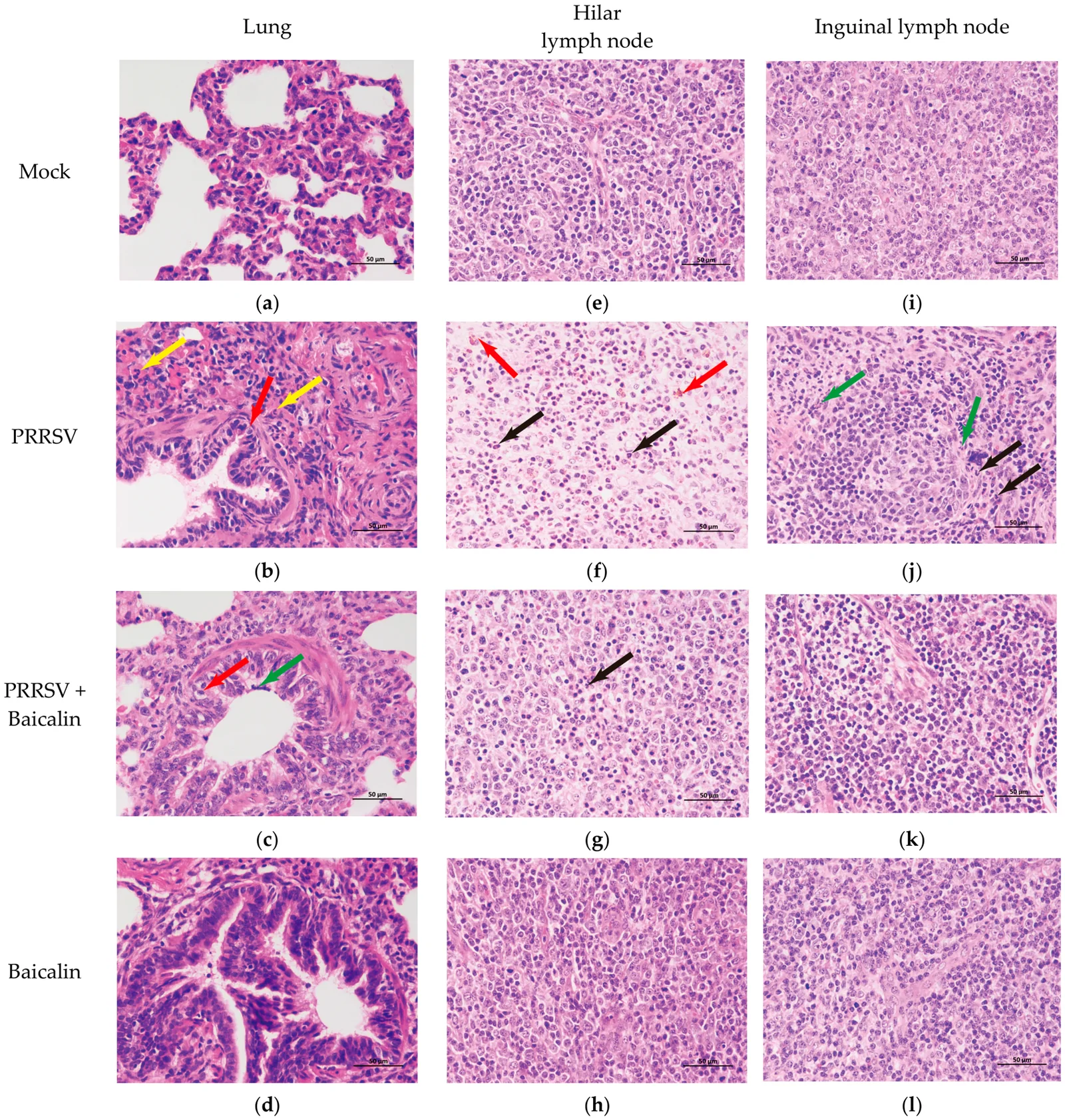
Representative histopathological findings in the lungs and lymph nodes of PRRSV-infected piglets receiving prophylactic dietary baicalin supplementation. (**a**,**e**,**i**) Mock group; (**b**,**f**,**j**) PRRSV group; (**c**,**g**,**k**) PRRSV plus baicalin group; and (**d**,**h**,**l**) baicalin-only group. (**a**–**d**) Lung; (**e**–**h**) hilar lymph node; and (**i**–**l**) inguinal lymph node. Yellow arrows indicate alveolar-septal thickening; red arrows indicate inflammatory-cell infiltration around bronchi or blood vessels; green arrows indicate focal disruption of tissue architecture; and black arrows indicate loosened lymphoid architecture and reduced lymphocyte density. Enlarged regions of interest are provided to facilitate visualization of the indicated lesions. Scale bar = 50 μm.

Histopathological changes in the lungs, hilar lymph nodes, and inguinal lymph nodes were further evaluated by H&E staining (Figure 7). Lung tissue from Mock animals showed intact alveolar architecture, open alveolar spaces, thin alveolar septa, and minimal inflammatory-cell infiltration around bronchi. Lymph-node architecture was preserved with an even distribution of lymphocytes. In contrast, PRRSV-infected lungs exhibited typical interstitial pneumonia, characterized by marked thickening of alveolar septa, narrowing of alveolar spaces, extensive inflammatory-cell infiltration around bronchi and blood vessels, and focal disruption of tissue architecture. Hilar lymph nodes showed loosened lymphoid architecture, reduced lymphocyte numbers, focal cellular degeneration, and vacuole-like changes. Similar disorganization and focal lymphocyte depletion were observed in inguinal lymph nodes.

Histopathological injury was attenuated in the PRRSV plus baicalin group. Alveolar architecture was better preserved, with less septal thickening and inflammatory-cell infiltration, although mild peribronchial infiltration remained focally. Hilar lymph nodes showed improved tissue organization and less lymphocyte depletion, and inguinal lymph nodes displayed a more uniform lymphocyte distribution with fewer focal lesions. Lung and lymph-node morphology in the baicalin-only group was comparable to that in the Mock group. These findings indicate that baicalin alleviated PRRSV-associated pulmonary and lymphoid tissue injury without causing overt histopathological abnormalities when administered alone.

**Figure 8:**
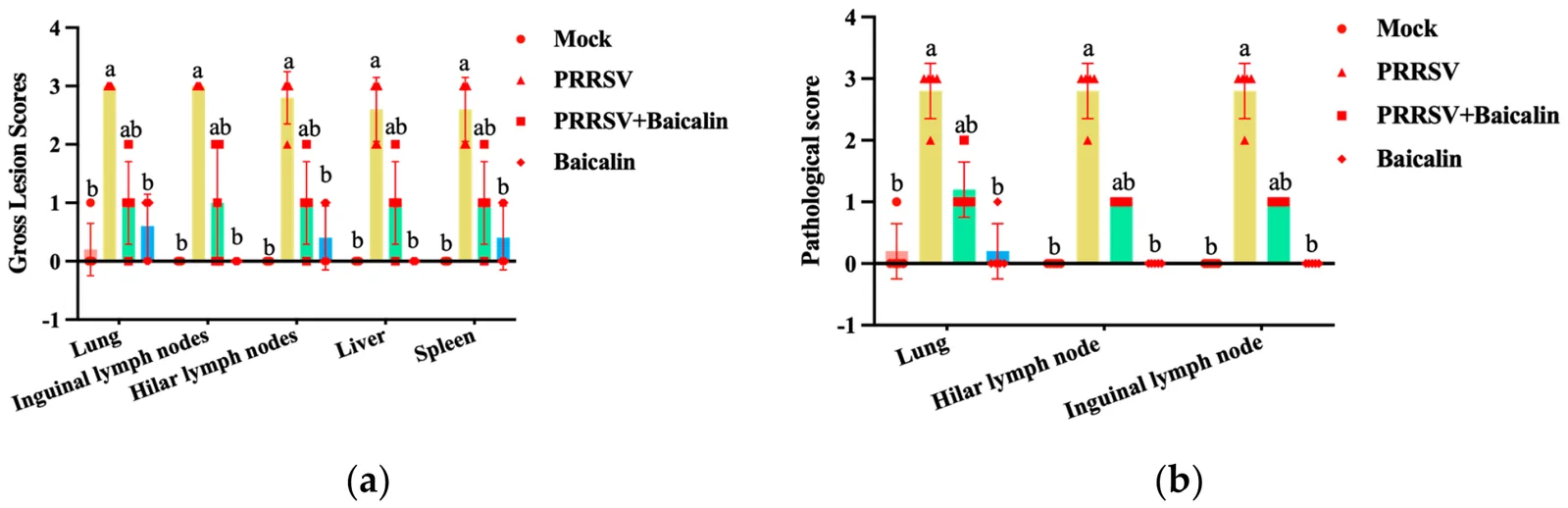
Effects of prophylactic dietary baicalin supplementation on gross and histopathological lesion scores in PRRSV-infected piglets. (**a**) Gross lesion scores for the lung, inguinal lymph node, hilar lymph node, liver, and spleen. (**b**) Histopathological scores for the lung, hilar lymph node, and inguinal lymph node. Light red, yellow, green, and blue bars represent the Mock, PRRSV, PRRSV plus baicalin, and baicalin-only groups, respectively. Red circles, triangles, squares, and diamonds indicate individual animal values in the Mock, PRRSV, PRRSV plus baicalin, and baicalin-only groups, respectively. Individual animal values (*n* = 5 piglets per group) are shown together with the median and interquartile range. Differences among groups were analyzed using the Kruskal–Wallis test followed by Dunn’s multiple-comparisons test. Groups that do not share the same lowercase letter are significantly different based on Dunn-adjusted *p* < 0.05, whereas groups sharing at least one letter are not significantly different.

As shown in Figure 8, gross lesion scores for the lung, inguinal lymph node, hilar lymph node, liver, and spleen were significantly higher in the PRRSV group than in the Mock group, and histopathological scores for the lung and both lymph nodes were likewise elevated. prophylactic dietary baicalin supplementation produced an overall downward trend in gross and histopathological lesion scores; however, differences from either the Mock or PRRSV group did not reach statistical significance. Scores in the baicalin-only group remained low and did not differ significantly from those in the Mock group. These results suggest that baicalin partially alleviated PRRSV-associated tissue injury and did not induce detectable pathological changes when administered alone.

### 3.7. Baicalin Modulates PRRSV-Induced Inflammatory Responses

**Figure 9:**
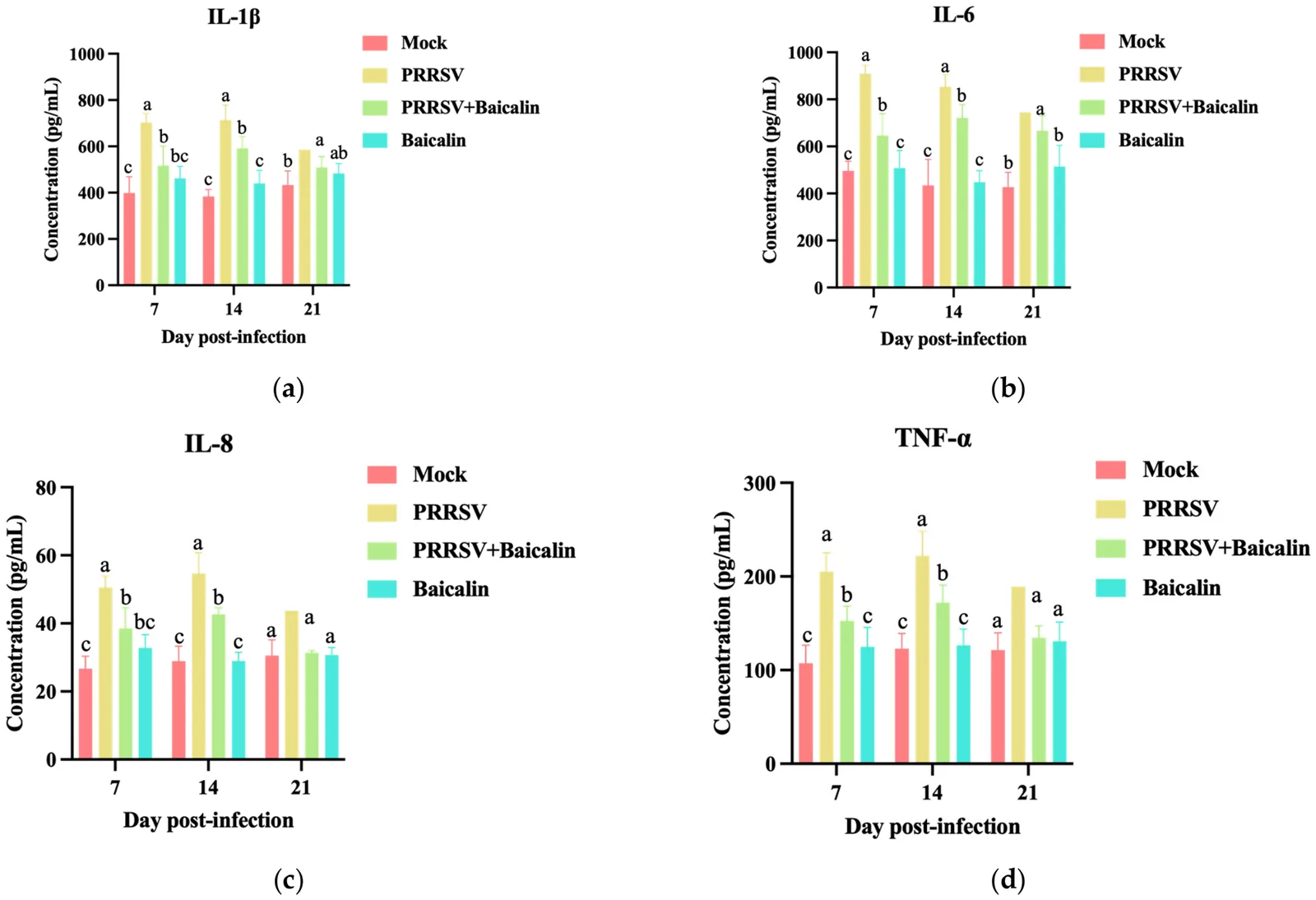
Effects of prophylactic dietary baicalin supplementation on serum pro-inflammatory cytokine concentrations in PRRSV-infected piglets. (**a**) IL-1β; (**b**) IL-6; (**c**) IL-8; and (**d**) TNF-α. Individual animal values (*n* = 5 piglets per group) are shown together with the mean ± SD. Data were analyzed using a mixed-effects model with group, time, and group-by-time interaction as fixed effects and individual piglet as the repeated-measures factor, followed by Šídák’s multiple-comparisons test for prespecified between-group comparisons at each time point. At each time point, groups that do not share the same lowercase letter are significantly different based on Šídák-adjusted *p* < 0.05, whereas groups sharing at least one letter are not significantly different.

Serum IL-1β, IL-6, IL-8, and TNF-α levels were measured to evaluate systemic inflammatory responses (Figure 9). All four pro-inflammatory cytokines increased after PRRSV infection, with significantly higher concentrations than in the Mock group on days 7 and 14, demonstrating a pronounced systemic inflammatory response. Cytokine levels were lower at multiple time points in the PRRSV plus baicalin group than in the PRRSV group; all four cytokines were significantly reduced on days 7 and 14.

An exploratory covariance analysis was performed to investigate whether the lower cytokine concentrations observed in the PRRSV plus baicalin group were associated with reduced viral burden. At both days 7 and 14, the PRRSV plus baicalin group showed lower serum viral loads and lower concentrations of IL-1β, IL-6, IL-8, and TNF-α than the PRRSV group. In day-specific ANCOVA models including contemporaneous log_10_-transformed serum viral load as a covariate, the adjusted group effects did not reach statistical significance for any of the four cytokines (all *p* > 0.05).

However, serum viral loads showed no overlap between the two infected groups, resulting in strong collinearity between group allocation and viral load. Consequently, the adjusted estimates were unstable and relied on extrapolation beyond the observed covariate ranges. These exploratory analyses could not reliably distinguish a direct group-associated effect from a secondary reduction in cytokine concentrations related to lower viral burden.

## 4. Discussion

The present study demonstrates that baicalin exerts coordinated antiviral and anti-inflammatory effects against porcine reproductive and respiratory syndrome virus (PRRSV). In primary porcine alveolar macrophages (PAMs), baicalin reduced infectious virus production and PRRSV nsp9 abundance in a concentration-dependent manner without evident cytotoxicity at concentrations up to 80 μg/mL. Stage-resolved assays localized the principal detectable antiviral activity to viral attachment, whereas direct virucidal activity and effects on entry, intracellular replication, and progeny release were not significant under the tested conditions. In piglets, dietary baicalin was associated with lower viremia and respiratory shedding, improved clinical outcomes, attenuation of pulmonary and lymphoid lesions, and reduced circulating IL-1β, IL-6, IL-8, and TNF-α. Collectively, these findings indicate that baicalin was associated with antiviral activity during the attachment phase in vitro and with lower viral burden and inflammatory cytokine concentrations in vivo. However, whether these in vitro and in vivo observations are mechanistically linked remains to be determined.

The attachment-specific activity is biologically relevant because successful PRRSV infection depends on interactions between viral envelope proteins and multiple attachment or entry factors on susceptible cells. Heparan sulfate facilitates initial attachment, whereas sialoadhesin/CD169 promotes virion binding and internalization in PAMs. CD163 is indispensable for productive infection and functions predominantly during internalization, membrane fusion, and uncoating rather than initial attachment [9,25]. The viral GP5/M complex interacts with CD169 in a sialic-acid-dependent manner, providing a defined molecular interface involved in PRRSV attachment and uptake [26]. Interference with this early phase can reduce the number of macrophages in which productive infection is established before viral RNA synthesis begins.

The attachment-stage results do not establish whether baicalin acts on a viral envelope protein or a cellular attachment factor. This distinction is important because baicalein, the aglycone of baicalin, was reported to bind PRRSV directly, inhibit EGFR phosphorylation and downstream PI3K–AKT signaling, and enhance immune responses in infected piglets [20]. These findings define the experimentally demonstrated activities of baicalein but cannot be directly extrapolated to baicalin because glucuronidation alters molecular polarity, membrane permeability, and protein-binding properties. Molecular binding and receptor-dependency assays are therefore required before a specific attachment target can be assigned to baicalin.

The reduction in serum viral loads provides a biologically plausible explanation for the improved clinical outcomes. PRRSV preferentially infects macrophage populations and disseminates through blood and lymphoid tissues; sustained viremia is consequently associated with continued tissue infection, immune dysfunction, and respiratory disease [4]. Lower PRRSV copies in serum and oropharyngeal and nasal swabs indicated reduced systemic viral burden and respiratory shedding. Limiting early viral dissemination would decrease the number of infected macrophages and the duration of virus-associated tissue injury, although the present experimental design did not formally establish mediation between viral-load reduction and each clinical endpoint.

An effect confined to the early phase of infection could nevertheless influence subsequent disease progression through cumulative effects over successive rounds of viral replication. A reduction in the number of initially infected macrophages may result in fewer virus-producing cells during the first replication cycle, thereby limiting the number of virions available to initiate subsequent cycles of infection and dissemination. Under this model, a relatively modest reduction in the establishment of infection could produce a larger difference in systemic viral burden over time. However, the present data do not demonstrate that interference during the attachment phase alone accounted for the observed differences in the piglets, and other direct or host-mediated effects of baicalin cannot be excluded. Immune responses may also contribute to the divergence in disease progression between the two infected groups. Early PRRSV replication has been associated with reduced natural killer cell-mediated cytotoxicity, altered circulating T-cell populations, and changes in innate cytokine production, which may contribute to delayed development of adaptive immunity [27]. In addition, PRRSV infection is characterized by relatively weak type I interferon responses and viral antagonism of interferon-associated signaling, potentially allowing early viral replication to proceed with limited antiviral restriction [28]. It is therefore plausible that reducing the initial viral burden could shorten or lessen the period of virus-induced immune dysregulation, allowing endogenous innate and cellular immune responses to contribute more effectively to subsequent viral control. This interpretation remains hypothetical because type I interferons, interferon-stimulated genes, natural killer cell activity, PRRSV-specific T-cell responses, and neutralizing antibodies were not measured in the present study. These variables should be evaluated in future studies to determine whether the in vivo effects associated with baicalin involve preservation or restoration of antiviral immunity in addition to reduced viral burden. Moreover, given the small group size, the observed survival proportions (4/5 versus 1/5) should be considered preliminary descriptive evidence rather than definitive proof that interference during viral attachment improves survival.

Natural products have previously shown protective activity in experimental PRRSV infection. *Cryptoporus volvatus* extract reduced serum viral loads and increased survival in piglets challenged with highly pathogenic PRRSV, with antiviral effects involving virus entry and RNA-dependent RNA polymerase activity [29]. More recently, hyperoside reduced PRRSV replication, inflammatory cytokine expression, and pathological injury through regulation of TLR4/NF-κB and p62/Nrf2/Keap1 signaling [30]. In mechanistic terms, this multistage activity may remain effective after infection has already been established, whereas an effect confined to the attachment phase would be expected to depend more strongly on compound availability at the time of initial virus–cell contact. Conversely, interference before productive infection is established could theoretically reduce the number of initially infected macrophages without requiring intracellular accumulation of the compound.

PRRSV-associated disease is driven not only by virus replication but also by dysregulated inflammatory signaling. PRRSV induces mitochondrial dysfunction, cytosolic mitochondrial DNA stress, and reactive oxygen species accumulation in PAMs, activating NF-κB and the NLRP3 inflammasome and promoting IL-1β production [18]. More recent evidence shows that PRRSV-2 nsp2 interacts with NLRP3 and promotes IKKβ-dependent NLRP3 translocation and oligomerization, providing a direct mechanistic link between a viral protein, inflammasome activation, and pulmonary inflammatory injury [31]. Earlier studies also connected PRRSV-induced IL-1β expression with TLR4/MyD88-dependent NF-κB, ERK1/2, and p38 MAPK signaling.

The reductions in IL-1β, IL-6, IL-8, and TNF-α observed here are therefore biologically consistent with attenuation of PRRSV-driven NF-κB/MAPK priming and NLRP3-dependent inflammatory amplification. Baicalin has been widely reported to regulate NF-κB, MAPK, oxidative stress, and inflammasome signaling in inflammatory disease models [10]. The lower cytokine concentrations observed in the PRRSV plus baicalin group on days 7 and 14 coincided with markedly lower serum viral loads. In the exploratory day-specific covariance analyses, the adjusted group effects did not reach statistical significance after contemporaneous viral load was included as a covariate. This pattern is consistent with the possibility that the lower cytokine concentrations were at least partly secondary to reduced viral burden. However, viral loads showed no overlap between the two infected groups, producing strong collinearity between group allocation and viral load and limiting the reliability of the adjusted estimates. Therefore, the present analysis cannot establish that the cytokine differences were entirely mediated by viral-load reduction, nor can it demonstrate a direct immunomodulatory effect of baicalin.

Baicalin should be considered a potential adjunct to vaccination and integrated herd management rather than a replacement for vaccines. Several studies have examined Chinese herbal preparations or defined plant-derived compounds as supportive interventions against PRRSV [32]. Astragalus polysaccharides have been associated with suppression of HP-PRRSV replication and protection of the pulmonary endothelial glycocalyx [33], whereas Isatis root polysaccharides inhibit PRRSV proliferation and regulate apoptosis and inflammatory responses in porcine macrophage-derived cells [34]. Unlike these polysaccharides, baicalin is a chemically defined flavone glycoside, and the present stage-specific experiments localized its principal detectable activity to attachment. Direct comparative studies using identical viral strains, cell systems, doses, and outcome measures are still required to determine whether these compounds provide complementary or overlapping effects.

An important limitation of the present animal experiment is that baicalin administration was initiated 2 days before PRRSV challenge. This prophylactic regimen represents a favorable experimental condition and does not reflect the common production scenario in which an intervention is initiated after infection or after clinical signs have emerged. Because a post-challenge administration group was not included, the present study cannot determine whether baicalin is effective against an established PRRSV infection. Accordingly, the in vivo findings should be interpreted as proof-of-concept evidence for prophylactic dietary supplementation rather than evidence of therapeutic efficacy. Future studies should include treatment initiated at defined intervals after challenge or after virological or clinical confirmation of infection and should evaluate dose–response relationships, therapeutic windows, and clinically relevant outcomes.

Further development should address oral exposure, antiviral breadth, and molecular target identification. Baicalin has limited oral bioavailability because of poor intestinal absorption, extensive metabolism, and enterohepatic processing; formulation strategies such as phospholipid complexes, nanoparticles, or other delivery systems have been proposed to improve systemic exposure [35]. Antiviral activity should also be evaluated against HP-PRRSV, NADC30-like, NADC34-like, and genetically divergent PRRSV-1 and PRRSV-2 strains. Quantitative binding methods and receptor-dependency assays will be necessary to determine whether baicalin interacts with the virion, cell-surface attachment factors, or both. The in vitro assessment was confined to PAMs and did not include MARC-145 cells. Stage-specific activity was evaluated against SCSN2020 without testing genetically diverse strains, and the animal experiment used a single dietary dose of 0.1 g/kg. These studies will determine whether the protective profile observed experimentally can be translated into a standardized adjunct for PRRS control.

## 5. Conclusions

This study demonstrates that baicalin exerts protective effects against PRRSV infection through complementary antiviral and anti-inflammatory activities. In PAMs, baicalin inhibited PRRSV infection in a concentration-dependent manner without evident cytotoxicity at concentrations up to 80 μg/mL. Stage-specific assays identified viral attachment as the principal phase affected, whereas no significant direct virucidal activity or inhibition of viral entry, intracellular replication, or progeny release was detected. In PRRSV-infected piglets, dietary baicalin supplementation reduced viremia and respiratory viral shedding, alleviated fever and clinical signs, mitigated impaired weight gain, improved survival, and attenuated pulmonary and lymphoid tissue injury. Baicalin also suppressed PRRSV-induced increases in IL-1β, IL-6, IL-8, and TNF-α. Collectively, these findings support baicalin as a promising feed-administered adjunct for PRRSV control. Further studies should identify its direct molecular target during viral attachment, optimize its oral bioavailability and formulation, and evaluate its efficacy against genetically diverse PRRSV strains under commercial production conditions.

## Figures and Tables

**Table 1:** Primers used for RT-qPCR.

Primer	Sequence (5′–3′)	Product Size (bp)
PRRSV-F	CTCCTGCGTTCTGCGGTTCCTTC	119
PRRSV-R	TCCCAAAGCGTGCCATCAATCCC	
β-actin-F	CATCACCAACTGGGACGACA	121
β-actin-R	GTTGGCCTTAGGGTTCAGGG	

## Data Availability

The original contributions of this study are included in the article; further inquiries can be directed to the first authors.
